# Lactylation omics of rabbit rotator cuff tear reveals differentially modified proteins and metabolic relating therapy targets

**DOI:** 10.3389/fmed.2026.1797466

**Published:** 2026-03-17

**Authors:** Tong Pan, Zhenlong Liu

**Affiliations:** 1 Department of Sports Medicine, Peking University Third Hospital, Institute of Sports Medicine of Peking University, Beijing, China; 2 Beijing Key Laboratory of Sports Injuries, Beijing, China; 3 Engineering Research Center of Sports Trauma Treatment Technology and Devices, Ministry of Education, Beijing, China

**Keywords:** functional enrichment, lactate, lactylation, modification sites, motif, omics

## Abstract

Proteins exert biological functions not only depending on abundance but also on regulation. Lactylation, a novel post-translational modification, can mediate metabolic reprogramming and epigenetic regulation, playing a crucial role in signal transduction, gene expression and cellular metabolism. Lactylation is also involved in various diseases, such as tumors, Alzheimer’s disease, heart failure and myocardial infarction. However, there is little research in musculoskeletal system. In this study, we conducted lactylation omics on rabbit rotator cuff tear samples and identified 2,624 modification sites on 851 proteins. We obtained results on subcellular localization, differentially modified proteins and functional pathway enrichment. Basing on motif, we proposed the “lysine co-lactylation modification effect” concept. Overall, lactylation mainly localized in cytoplasm, mitochondria and nucleus, with its functions enriching in RNA processing, DNA processing and cellular metabolism. Considering that lactylation is widely present and significantly occurs in rotator cuff tears, we aim to identify the key targets through which lactylation exerts its effects and to intervene it, ultimately providing new insights and therapeutic approaches for clinic therapy.

## Graphical abstract

**Figure:**
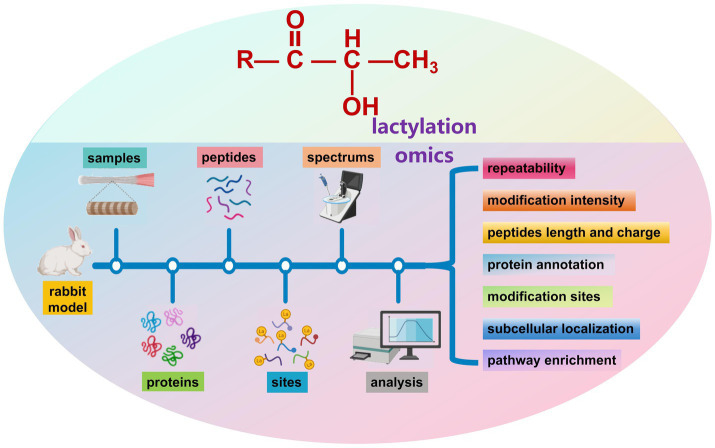

## Highlights

- Lactate and lactylation levels significantly increase in rotator cuff tear.
- Motifs indicate “lysine co-lactylation modification effect” at lactylation sites.
- Lactylation functionally enriches in gene expression and cellular metabolism pathways.

## 1 Introduction

The rotator cuff refers to a group of muscles around the shoulder joint, including supraspinatus, infraspinatus, teres minor and subscapularis muscles, which assist movement and maintain stability. Rotator cuff tears often occur at the junction of the supraspinatus tendon ends and the humeral head, namely tendon-bone interface. It usually presents with shoulder pain and limited motion, often leading to osteoarthritis (1). Its incidence increases with age, approximately 13% at the age of 50, 20% at 60, 31% at 70 (2). Due to ischemia and hypoxia, the healing capacity of rotator cuff is poor, and clinical treatment effect is also usually limited (3–5). Under conservative treatment, the torn tendons are difficult to reconnect and often continue to enlarge. Surgery mainly involves suturing and fixing the torn ends to the bone surface to achieve reconnection (6, 7). However, the mechanical properties of the regenerated tissues are poor, and the re-tear rate is as high as 25–75% (8, 9). Therefore, in-depth research on the molecular biological mechanisms is urgently needed.

Life processes depend not only on protein abundance but also on modification. Post-translational modifications (PTMs) can regulate protein activity and interaction, making modification omics pivotal for elucidating life activity mechanisms, identifying disease markers, and discovering drug targets. In 2019, Zhang D first reported lysine lactylation (Kla), a landmark discovery in novel acylation modifications (10). Upon M1 polarization, macrophages shift metabolism from oxidative phosphorylation (OXPHOS) to glycolysis, leading to lactate accumulation and increased H3K18la, which induced the expression of homeostatic genes (such as arginase, *Arg1*). Notably, H3K18la results in spontaneous macrophage polarization from pro-inflammatory M1 phenotype to anti-inflammatory M2 phenotype, thereby halting M1-mediated inflammation, restoring homeostasis, and promoting tissue repair. This finding suggested that lactate metabolic signals can be transduced into gene expression signals.

Lactylation can affect the structure and function of proteins, playing a crucial role in signal transduction, gene expression and cellular metabolism, thus participating in the occurrence and development of various diseases, such as tumors (11–14), myocardial infarction (15), heart failure (16), myopia (17), ferroptosis (18), and autophagy (19). Therapy targeting lactylation intervention represents a brand new direction. In breast cancer (20), lactate mediated MRE11-K673la leads to drug resistance by enhancing homologous repair recombination, while LDH inhibitor can improve chemotherapy sensitivity. In pancreatic ductal adenocarcinoma (20), “LDHA-H3K18la-TTK/BUB1B-LDHA” positive feedback centered around H3K18la exacerbates the malignant proliferation, while inhibiting its “writer” p300 can precisely disrupt it. In Alzheimer’s disease (21), “PKM-H4K12la-PKM” positive feedback centered around H4K12la worsens the condition, while pyruvate kinase (PKM) inhibitor sodium dichloroacetate (DCA) can improve it. In innate immunity (22), cGAS-K156la inhibits the immune and inflammatory responses mediated by “cGAS-STING” signaling pathway, while monocarboxylate transporter (MCT) inhibitor can prevent lactate uptake and thus reverse immune suppression.

Given that lactylation has a special effect on microenvironment homeostasis and injury repair (10), and other studies showing elevated lactate levels under conditions such as exercise, injury, and infection (23–25). Therefore, we hypothesize that after rotator cuff tear or surgery, the levels of lactate and lactylation should also increase, and may help tissue repair. To verify it, we collected clinical human tendon samples for lactate and lactylation detection, and constructed a rabbit rotator cuff tear model for lactylation modification omics.

We aim to deeply explore the molecular biological changes in rotator cuff tear, hoping to find key targets and regulatory mechanisms of lactylation, and ultimately provide new strategies for clinical treatment strategies.

## 2 Results

### 2.1 Lactate and lactylation levels increase in human injured tendon samples

**Figure 1:**
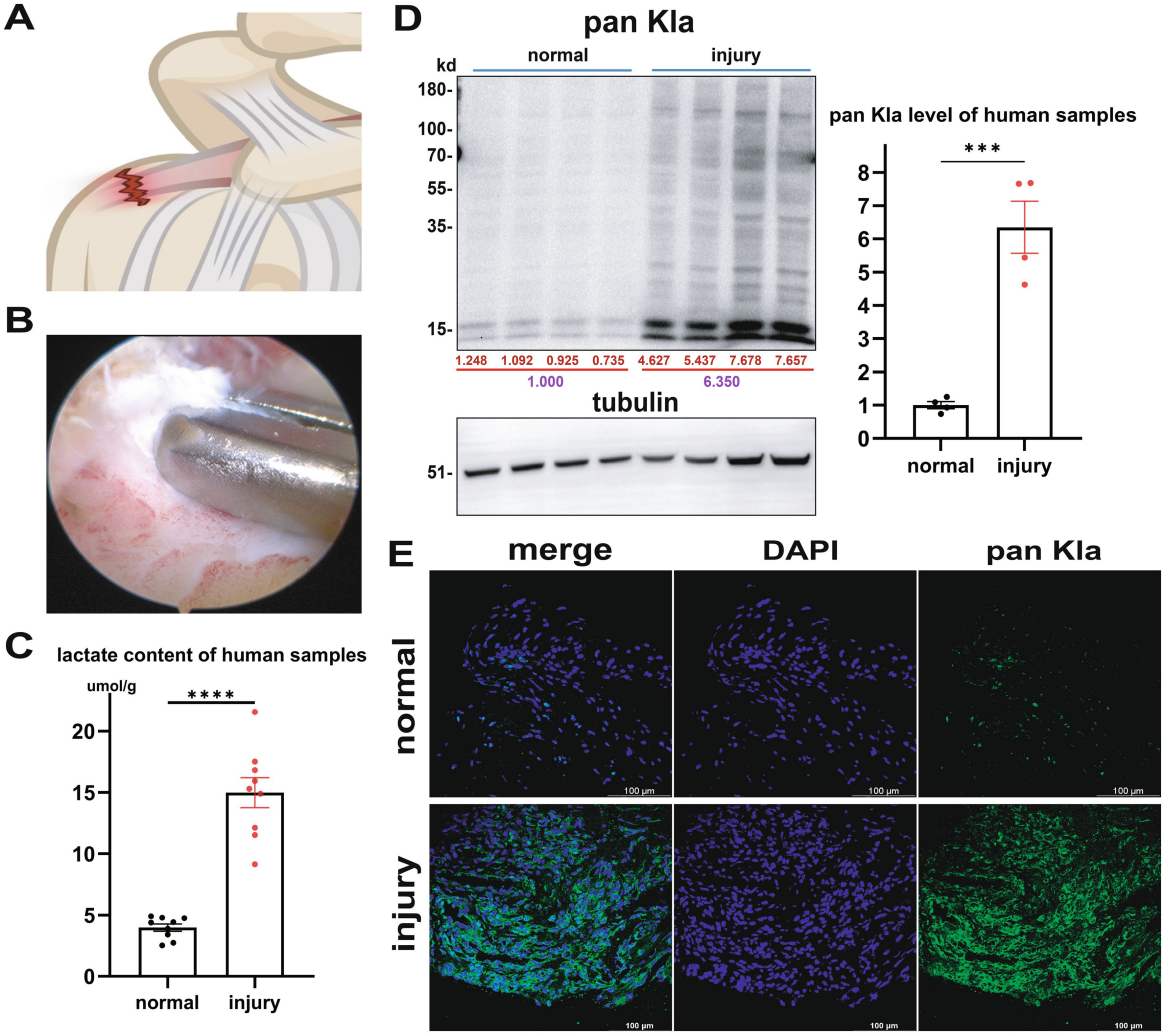
Detection of lactate and lactylation in human tendons. **(A)** Illustration of Rotator Cuff Tear. **(B)** Collecting human samples. **(C)** Lactate content of human samples (*n* = 9, *p* < 0.001). **(D)** WB pan Kla results of normal and injury samples with quantification of relative levels (*n* = 4, *p* < 0.001). **(E)** IF results of pan Kla demonstrated a more significant presence in injury group.

Rotator cuff tears often occur at the junction of the supraspinatus tendon ends and the humeral head (Figure 1A). With ethical approval, we collected clinical samples from the Department of Sports Medicine at Peking University Third Hospital (Figure 1B), including damaged and undamaged tendons.

Lactate content measurement showed that the lactate content was higher in the injury group (Figure 1C). The average in the ctr group was 3.971 ± 0.898 μmol/g, while the injury group was 14.980 ± 3.681 μmol/g (*n* = 9, *p* < 0.001). WB pan Kla detection results showed that (Figure 1D), setting average level of normal group as 1.000, the injury group was 6.350 (*n* = 4, *p* < 0.001). After stripping, tubulin was incubated. Immunofluorescence results (Figure 1E) also showed that pan Kla was widely present and significantly increased in the injury group.

In summary, the significantly increased lactate and lactylation in injury samples suggest potential active role in repair process of rotator cuff tear, aligning with the known function in homeostasis maintenance and anti-inflammatory that lactylation promotes macrophage M2 polarization.

### 2.2 Establishment and lactylation detection of rabbit rotator cuff tear model

**Figure 2:**
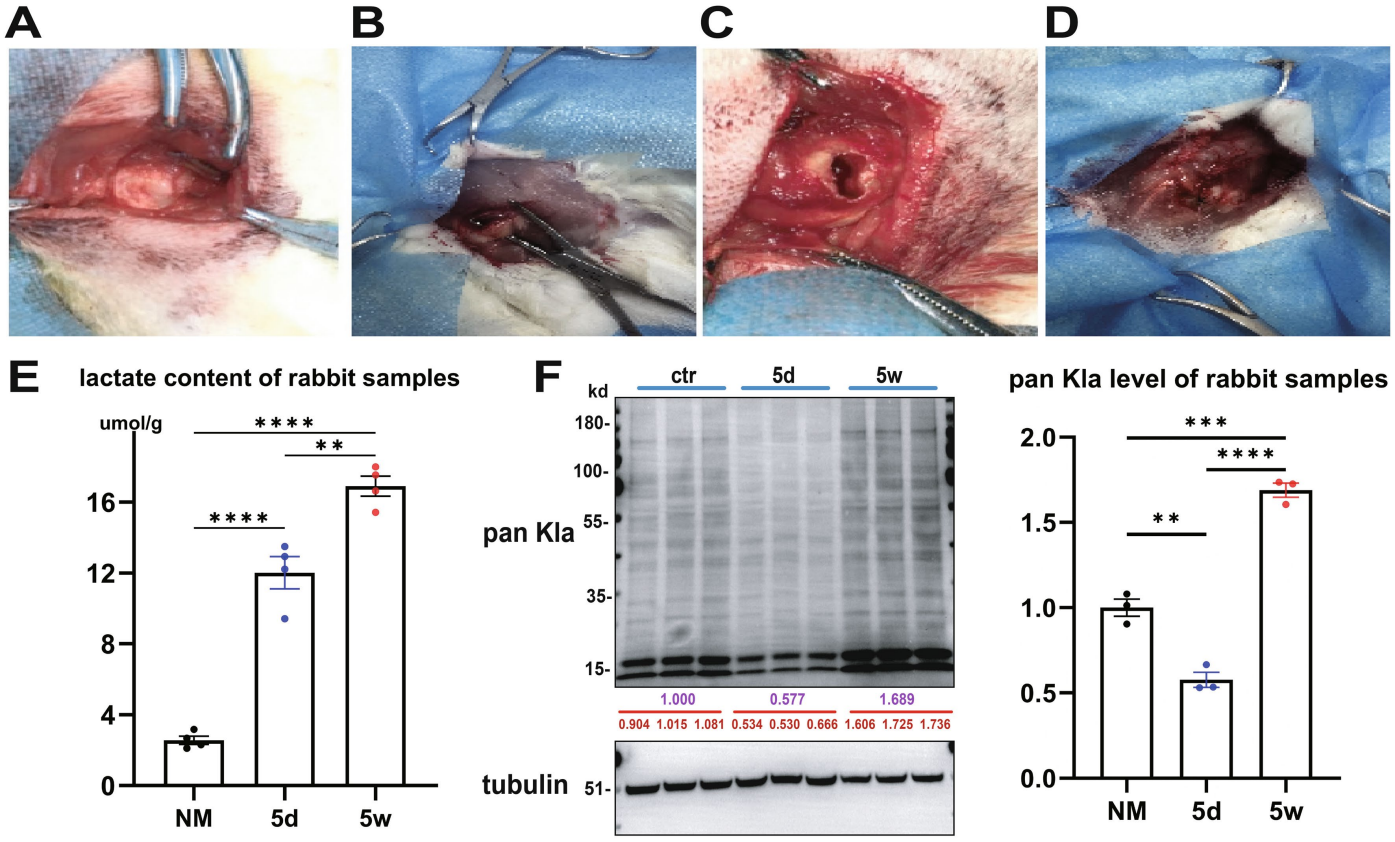
Establishment and lactylation detection of rabbit rotator cuff tear model. **(A–D)** Establishing re-sutured supraspinatus tendon rabbit rotator cuff tear model. Identification, isolation, transection, and suturing. **(E)** Lactate content of NM, 5d and 5w group rabbit samples (*n* = 4). **(F)** WB pan Kla level of NM, 5d and 5w group rabbit samples (*n* = 3).

We established a re-sutured supraspinatus tendon detachment rabbit rotator cuff tear model.

(Figures 2A–D). Studies have shown that the period from 0 to 7 days after tendon injury is the acute inflammatory phase with inflammatory cell infiltration and lactate accumulation. The period from 4 to 10 weeks is the chronic remodeling phase with collagen maturation and tissue re-shaping (26–28). Therefore, we focused on normal (NM) and time points of 5 days (5d) and 5 weeks (5w) after surgery, and collected samples for detection.

Lactate content (Figure 2E) was 2.565 ± 0.4652 μmol/g in NM group, 12.516 ± 2.135 μmol/g in 5d group and 20.386 ± 1.700 μmol/g in 5w group (*n* = 4). WB, pan Kla detection (Figure 2F) results showed that, setting the average value of NM group as 1.000, the 5d group was 0.577 and the 5w group was 1.689.

These results demonstrated that both lactate and lactylation showed an overall increasing trend, but the intermediate processes were different: lactate continued to increase, while lactylation initially decreased but then increased. This strange contradictory phenomenon is worthy of exploration.

### 2.3 Detection and overall analysis of lactylation modification omics

To clarify molecular changes, we conducted lactylation modification omics on the rabbit samples of the NM, 5d and 5w group (*n* = 3), and obtained results including modification intensity, differentially modified proteins and sites, subcellular localization, motifs and enrichment pathways.

**Figure 3:**
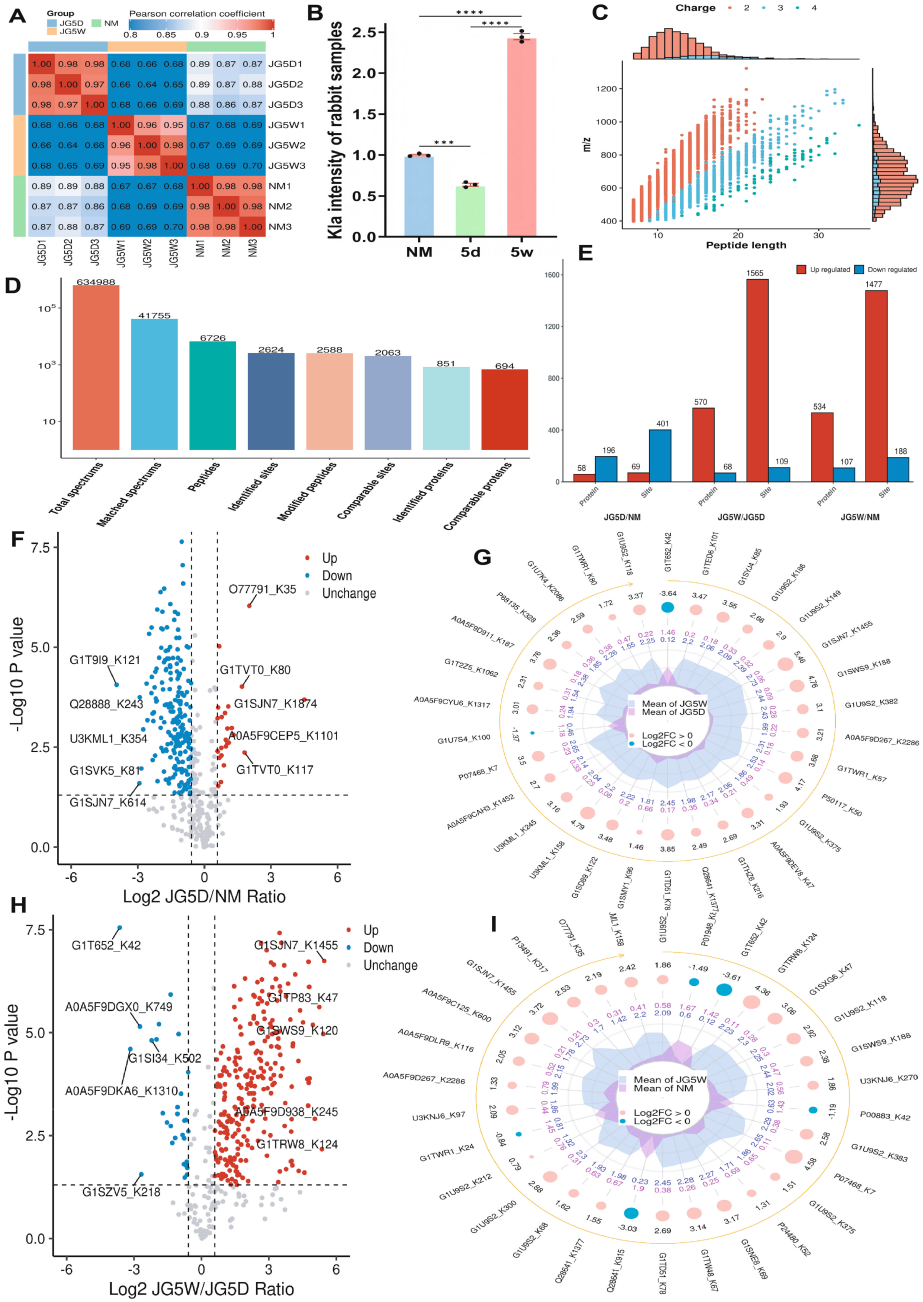
Overall analysis of lactylation omics. **(A)** Repeatability analysis of samples using Pearson’s correlation coefficient. **(B)** Relative quantitative analysis of lactylation. **(C)** Analysis of identified peptide segments, including length and charge. **(D)** All identified spectra, peptide segments, sites, and proteins. **(E)** Differentially modified proteins and sites. **(F,G)** Differentially modified sites between the 5d group and the NM group. **(H,I)** Differentially modified sites between the 5w group and the NM group.

Regarding the quality of the samples, the Pearson’s Correlation Coefficient analysis indicated good intra-group repeatability (Figure 3A). The relative intensity quantitative showed that (Figure 3B), with the average value of the NM group set as 1.000, the 5d group was 0.638 and the 5w group was 2.445. It was consistent with the previous WB results that the change trends of lactate and lactylation were inconsistent.

Among the 6,726 identified peptides, most carried 2–3 charges and had a length of 7–20 amino acids, which conformed to the general rules based on enzymatic digestion and mass spectrometry fragmentation methods (Figure 3C). A total of 2,624 modification sites and 851 modified proteins were identified, among which 2063 and 694 were comparable, respectively, (Figure 3D). In addition, we counted the differentially modified proteins and sites (Figure 3E). Among the differentially modified proteins compared with the NM group, 58 were upregulated and 196 were downregulated in the 5d group, while 534 were upregulated and 107 were downregulated in the 5w group (Figures 3F,H). Among the differentially modified sites compared with the NM group, 69 were upregulated and 401 were downregulated in the 5d group, and 1,477 were upregulated and 188 were downregulated in the 5w group (Figures 3G,I).

### 2.4 Subcellular localization of differentially lactylation modified proteins

Subcellular localization of proteins is intrinsically related to their biological functions. Mitochondrial proteins are mostly associated with cellular respiration and OXPHOS in energy metabolism, while nuclear proteins are mostly involved in gene expression.

**Figure 4:**
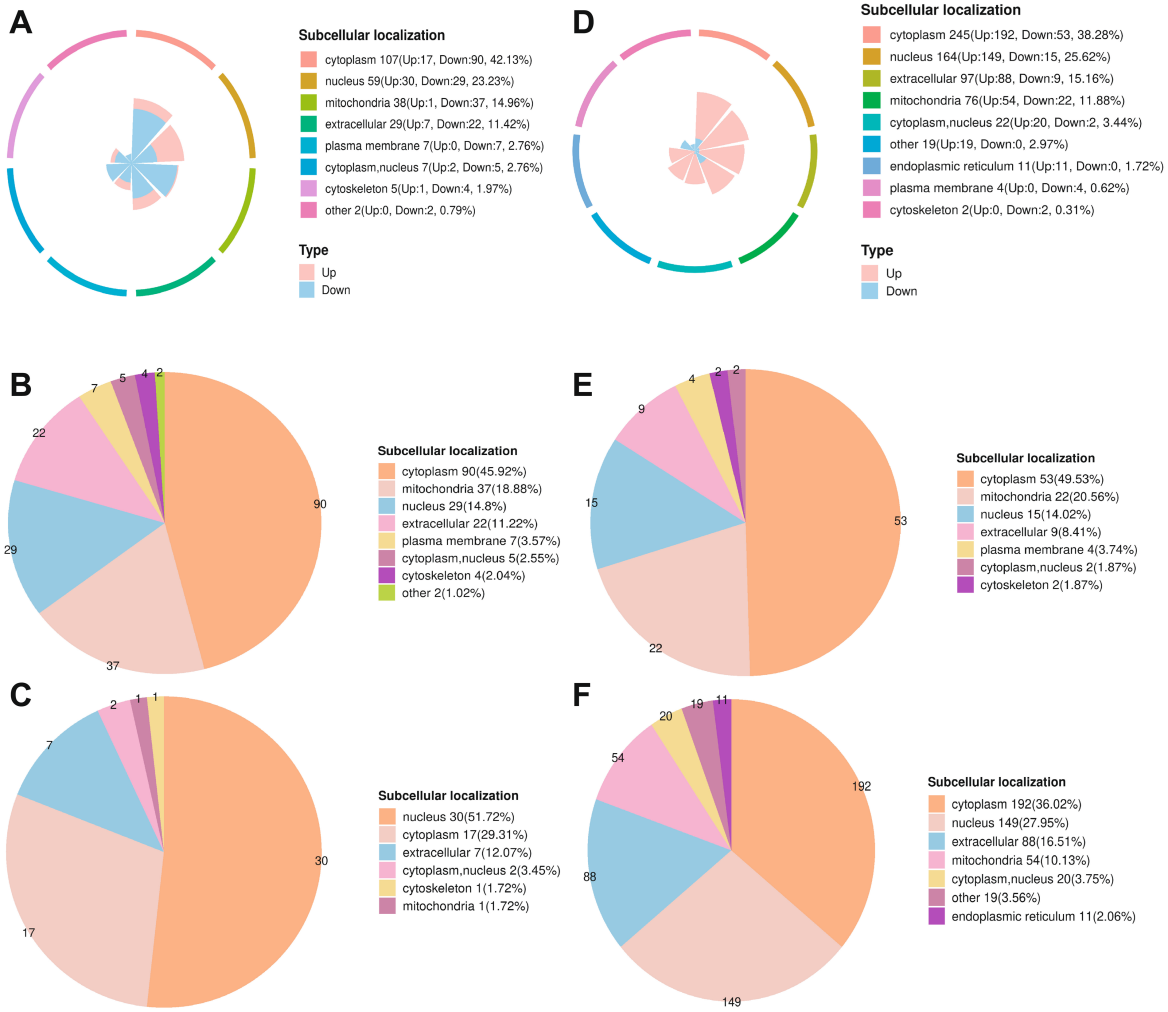
Subcellular localization of differentially modified sites. **(A–C)** Subcellular localization of differentially lactylation modified proteins in 5d group compared to NM group. **(A)** All differentially modified proteins in 5d group. **(B)** Down-regulated. **(C)** Up-regulated. **(D–F)** Subcellular localization of differentially lactylation modified proteins in the 5w group compared to the NM group. **(D)** All differentially modified proteins in 5w group. **(E)** Down-regulated. **(F)** Up-regulated.

Compared to the NM group, 254 differentially modified proteins were identified in 5d group (Figure 4A), with 107 (42.13%) in cytoplasm, 59 (23.23%) in nucleus, and 38 (14.96%) in mitochondria. Among the 196 down-regulated (Figure 4B), 90 (45.92%) in cytoplasm, 37 (18.88%) in mitochondria, and 29 (14.8%) in nucleus. Among the 58 up-regulated (Figure 4C), 30 (51.72%) in nucleus, and 17 (29.31%) in cytoplasm.

In 5w group, 640 differentially modified proteins were identified (Figure 4D), with 245 (38.28%) in cytoplasm, 164 (25.62%) in nucleus, 97 (15.16%) in extracellular, and 76 (11.88%) in mitochondria. Among the down-regulated (Figure 4E), 53 (49.53%) in cytoplasm, 22 (20.56%) in mitochondria, and 15 (14.02%) in nucleus. Among the up-regulated (Figure 4F), 192 (36.02%) in cytoplasm, 149 (27.95%) in nucleus, 88 (16.51%) in extracellular, and 54 (10.13%) in mitochondria.

The predominance of differentially modified proteins in the cytoplasm, nucleus, and mitochondria underscores their functional relevance to rotator cuff repair. Cytoplasmic proteins (accounting for around 40–50%) likely mediate metabolic reprogramming of cellular metabolism. Nuclear proteins (around 15–30%) may drive transcriptional activation of repair-related genes, such as extracellular matrix synthesis and anti-inflammatory factors. Mitochondrial proteins (around 10–20%) are pivotal for sustaining OXPHOS and ATP production, addressing the ischemia/hypoxia-induced healing impairment characteristic of rotator cuff tears.

Notably, the dynamic shifts in localization between 5d (acute phase) and 5w (chronic remodeling phase), such as increased nuclear up-regulation at 5w, reflect lactylation’s role in coordinating stage-specific repair programs: early modulation of cytoplasmic metabolism to manage inflammation, followed by nuclear-driven gene expression to guide long-term tissue regeneration. This compartmentalized regulation highlights lactylation as a versatile switch linking metabolic states to functional outputs in tendon healing.

### 2.5 Motif analysis and the “lysine co-lactylation modification effect”

Motifs refers to the specific amino acid residue sequence pattern that causes the mutual recognition and interaction between enzymes and substrates. In modification omics, motif serves as coordinate for modification sites and can help accurately reveal the targeting sites for modification enzymes, including “writers” and “erasers” (29, 30). For histones, specific transcription factors can recognize motif to regulate chromatin state and gene expression, closely relating epigenetic regulation network (31, 32).

Our omics identified 2,624 modification sites, and the 10 amino acids upstream and downstream of each site were used for motif analysis. When the number of a characteristic sequence is more than 20 and the *p* value is less than 0.000001, it is considered as a motif sequence. We designated the lactylated lysine (K) as position 0, and numbered the 20 upstream and downstream amino acids from −10 to 10. The log_10_ (*p* value) was used to represent the difference, with positive values indicating promotion of lactylation and negative values indicating inhibition.

**Figure 5:**
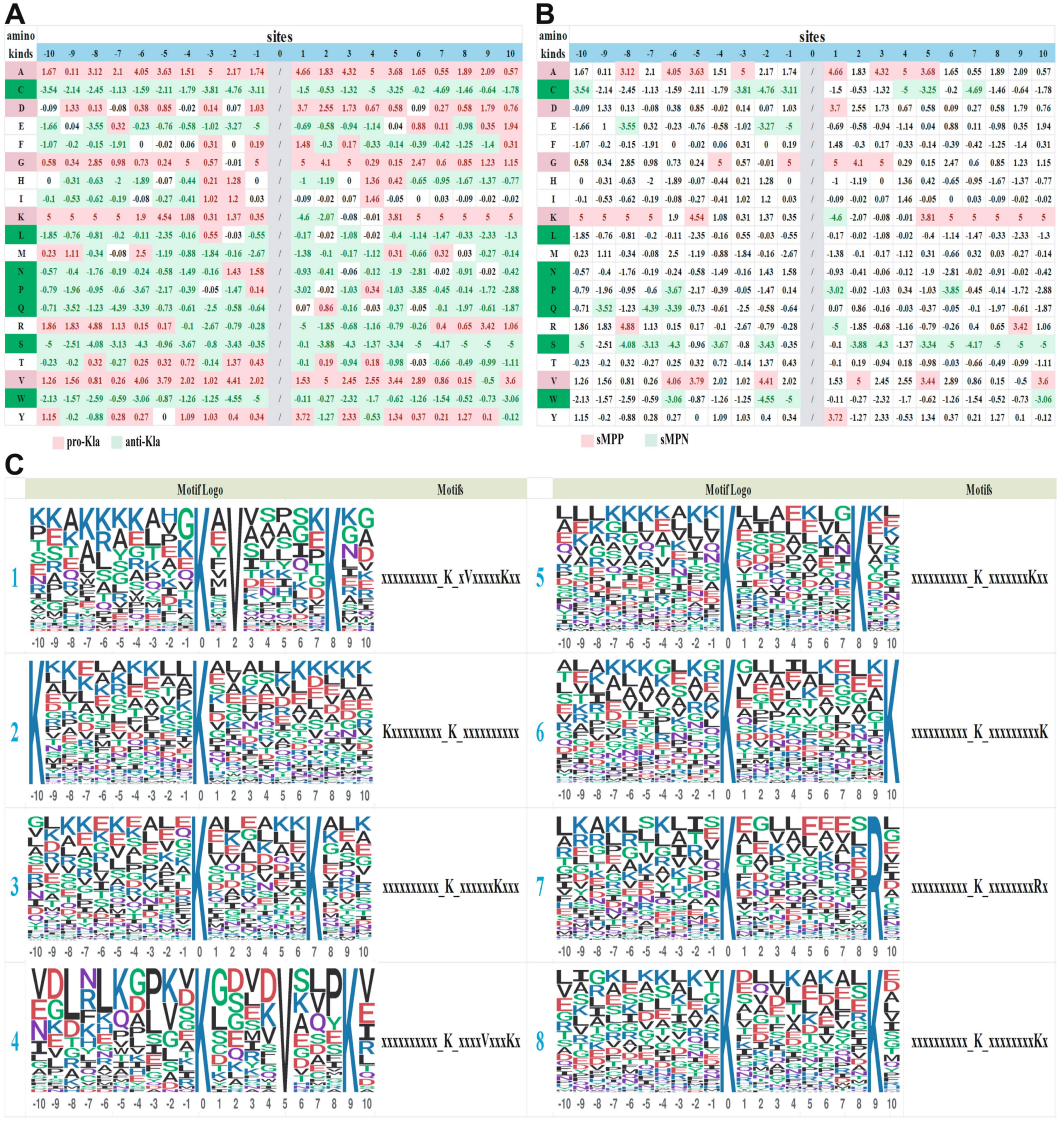
Motif analysis of lactylation modification. **(A)** The log10 *p* values of different amino acids at various sites, with values ≥ 0.1 filled in red while≤ − 0.1 filled in green. **(B)** sMPP and sMPN were screened based on the criteria of log10 p values ≥ 3 or ≤ − 3. **(C)** Top 8 Motifs sequences.

We drew a motif heat map of lactylation sites in rabbit rotator cuff tear, and classified sites with log_10_ (*p* values) ≥ 0.1 as promoting lactylation (pro-Kla) sites and those ≤ − 0.1 as anti lactylation (anti-Kla) sites (Figure 5A). We also classified amino acids into pro-Kla, anti-Kla and neutral according to their effects on lactylation (Table 1). The criteria were as follows: among the 20 sites upstream and downstream, amino acid sites with ≤ 2 anti-Kla and ≥ 15 pro-Kla sites were pro-Kla amino acids, and those with ≤ 2 pro-Kla and ≥ 15 anti-Kla sites were anti-Kla amino acids. For pro-Kla amino acids such as alanine (A), aspartate (D), glycine (G), and valine (V), regardless of their position, they always significantly promoted lactylation at the 0th K site. For anti-Kla amino acids such as cysteine (C), leucine (L), glutamine (Q), serine (S), and tryptophan (W), they always inhibited the lactylation modification at the 0th K site.

**Table 1:** Classification of amino acid types and their impact on 0th lysine site.

Classification	Type		pro-Kla site		anti-Kla site		Neutral site	
			Number	Percentage	Number	Percentage	Number	Percentage
pro-Kla amino acid	Alanine	A	20	100%	/	/	/	/
	Aspartate	D	15	75%	/	/	5	25%
	Glycine	G	19	95%	/	/	1	5%
	Lysine	K	16	80%	2	10%	2	10%
	Valine	V	19	95%	1	5%	/	/
anti-Kla amino acid	Cysteine	C	/	/	20	100%	/	/
	Leucine	L	1	5%	16	80%	3	15%
	Asparagine	N	2	10%	15	75%	3	15%
	Proline	P	2	10%	16	80%	2	10%
	Glutamine	Q	1	5%	16	80%	3	15%
	Serine	S	/	/	20	100%	/	/
	Tryptophan	W	/	/	20	100%	/	/
Neutral	Glutamate	E	5	25%	13	65%	2	10%
	Phenylalanine	F	5	25%	12	60%	3	15%
	Histidine	H	4	20%	12	60%	4	20%
	Isoleucine	I	3	15%	6	30%	11	55%
	Methionine	M	5	25%	13	65%	2	10%
	Arginine	R	10	50%	10	50%	/	/
	Threonine	T	8	40%	11	55%	1	5%
	Tyrosine	Y	14	70%	5	25%	1	5%

/, no values.

From the perspective of log_10_ (*p* values), values closer to 5 represent stronger modification tendency enhancement (MTE), while those closer to −5 represent stronger modification tendency suppression (MTS). We defined significant modification tendency enhancement (sMTE) and significant modification tendency suppression (sMTS) as log_10_ (*p* value) ≥ 3 or ≤ − 3 (Figure 5B).

For example, for the 5th site, sMTE contains lysine (K, 3.81), alanine (A, 3.68) and valine (V, 3.44), while sMTS contains serine (S, −3.34) and cysteine (C, −3.25). For lysine (K) itself, sMTE occurs at positions −10, −9, −8, −7, +6, +7, +8, +9, +10 (all 5), −5 (4.54) and 5 (3.81), while sMTS only occurs at +1 (−4.6). Notably, when K is present at any position from −10 to −5 or +5 to +10, either upstream or downstream, the 0th K shows a significant strong lactylation preference. This symmetrical characteristic of the upstream and downstream implies that when two adjacent K sites are spaced 5–10 amino acid residues apart, both K sites have a strong lactylation modification preference. We name this phenomenon the “lysine co-lactylation modification effect”.

From the perspective of specific peptide sequences, we listed top 8 typical Motifs (Figure 5C). Taking example of Motifs 1, “xxxxxxxxxx_K_xVxxxxxKxx,” when the 2nd downstream site is valine (V) and the 8th downstream site is lysine (K), the 0th K site exhibits the most significant lactylation modification preference. Among them, Motifs 2 (Kxxxxxxxxx_K_xxxxxxxxxx), 3 (xxxxxxxxxx_K_xxxxxxKxxx), 5 (xxxxxxxxxx_K_xxxxxxxKxx), 6 (xxxxxxxxxx_K_xxxxxxxxxK) and 8 (xxxxxxxxxx_K_xxxxxxxxKx) all have a spacing of 5–10 amino acid residues between two K sites, supporting the “lysine co-lactylation modification effect” hypothesis.

### 2.6 Functional pathway enrichment of differentially modified proteins

**Figure 6:**
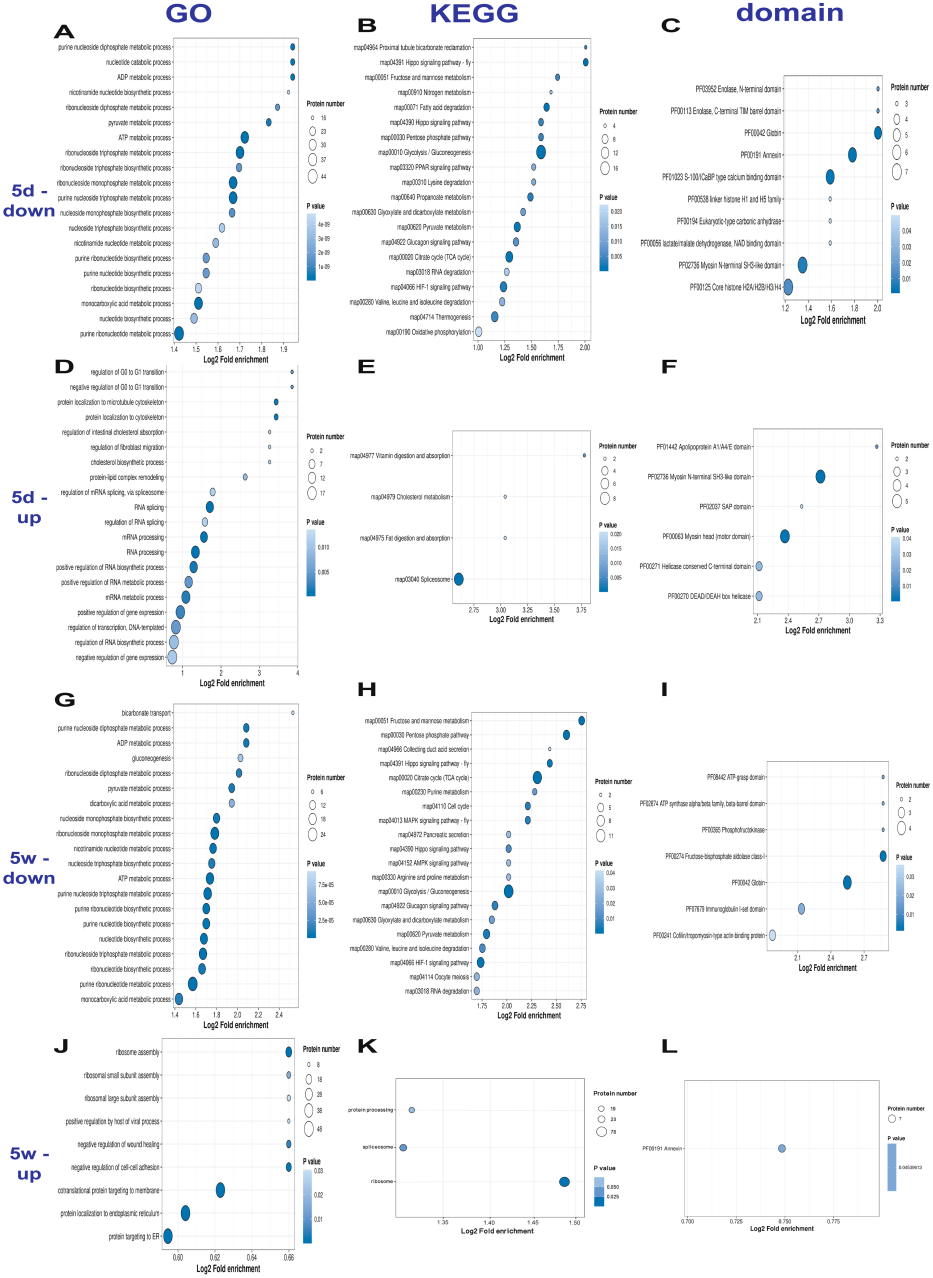
Functional Enrichment Pathways of Differentially Modified Proteins. **(A–C)** Down-regulated differentially modified proteins in 5d group compared to NM group. **(D–G)** Up-regulated differentially modified proteins in 5d group compared to NM group. **(H–J)** Down-regulated differentially modified proteins in 5w group compared to NM group. **(J–L)** Up-regulated differentially modified proteins in 5w group compared to NM group.

We performed enrichment analysis of Gene Ontology (GO), Kyoto Encyclopedia of Genes and Genomes (KEGG) and protein domains (Figure 6), with specific categories summarized (Supplementary Tables S1, S2).

Compared to NM group, the GO classification revealed that the most significantly down-regulated categories of 5d group included ATP, nucleoside and monocarboxylic metabolic process. While the most significantly up-regulated included RNA processing, DNA processing and gene expression regulation. In KEGG enrichment, the most significantly down-regulated included glycolysis/gluconeogenesis, TCA cycle, OXPHOS, while the most significantly up-regulated included spliceosome. In protein domains, the most significantly down-regulated included globin, annexin, S-100/ICa calcium-binding domains and core histones, while the most significantly up-regulated included myosin N-terminal and myosin head motor domain.

As for 5w group, in GO classification, the most significantly down-regulated included nucleotide and ATP metabolic process, while the most significantly up-regulated included ribosome assembly, wound healing and cell adhesion. In KEGG enrichment, the most significantly down-regulated pathways included glycolysis/gluconeogenesis, TCA cycle, pyruvate metabolism and HIF-1 signaling pathway, while the most significantly up-regulated included ribosome and spliceosome process. Among the protein structural domains, the most significantly down-regulated included aldolase and globin, while the most significantly up-regulated included annexin.

## 3 Discussion

### 3.1 Lactate content and lactylation level are inconsistent

In our rabbit rotator cuff tear model, there is a weird inconsistence in change trends of lactate and lactylation. Although most studies indicate that lactate enhances lactylation in concentration-dependent manner (33, 34), the specific process of lactylation is not merely a direct combination between lactate and lysine, but also depends on substrates, lactyl-CoA synthases, and regulatory enzymes.

Substrates for lactylation are lactyl-CoA (10) and lactoylglutathione (LGSH) (35), and the average concentration of lactyl-CoA of each cell in the wet weight of mouse heart tissue is around 1.14 × 10^−8^ pmol/Lg (36). Furthermore, two lactyl-CoA synthases have been identified, namely acetyl-CoA synthetase 2 (ACSS2) (37) and GTP-dependent succinyl-CoA synthetase (GTPSCS) (38). The regulatory enzymes for lactylation mainly include “writers” and “erasers.” “Writers” mainly include lysine acetyltransferases (KATs) and alanyl-tRNA synthetases (AARSs). KATs lack specificity (39) and participates in several modifications such as acetylation, lactylation (10), and propionylation (40) by forming acyl-CoA. Key lactylation-related KATs include: GCN5 (KAT2), CBP/p300 (KAT3A/3B) (33), TIP60 (KAT6) (41), and HBO1 (KAT7) (13). In contrast, the function of AARSs (11, 22, 42) differ from KATs that AARSs directly use lactate to complete lactylation without lactyl-CoA. “Erasers” mainly include histone deacetylases (HDACs) and sirtuins (SIRTs).

It is possible that changes in function of “writers,” “erasers” or lactyl-CoA synthases due to lactylation modifications might result in the inconsistent change trends. Our omic results show no significant changes in KATs, AARSs, SIRTs, ACSS2 or GTPSCS. However, HDAC1 is an exception. In NM group and 5w group, HDAC1-K412la was undetected due to absence or insufficient intensity, whereas it was detectable in 5d group. We hypothesize that HDAC1-K412la increases its “eraser” activity and leads to decreased lactylation level in 5d group.

This hypothesis can be supported by relevant research on HDACs and lactylation. On one hand, HDACs are indeed the primary eraser enzymes for lactylation, with effects approximately 1,000 times stronger than those of SIRTs (43, 44). On the other hand, HDACs themselves can also undergo lactylation modification, which affects their deacetylase function. Dong Q (45) found that HDAC1-K412/438la enhances its “eraser” activity but primarily further reduces H3K27ac levels, thereby leading to the silencing of target genes.

### 3.2 Lactylation modification omics warrants further exploration

Traditionally, lactate was considered a metabolic waste product of glycolysis. However, research has implicated it in “muscle-liver-muscle” Cori cycle (46), “astrocyte-neuron lactate shuttle” (47) and the Warburg effect (48). Moreover, lactate-mediated lactylation is an important post-translational modification. We conducted lactylation omics of rabbit rator cuff tear, and presented data on subcellular localization, motif and functional pathway enrichment. In-depth analysis will help us further understand the molecular biological changes and mechanisms after rotator cuff tear, ultimately discovering new targets for clinical therapy.

In motif, we list the effects of different amino acid on lactylation modification sites, including pro-Kla amino acids (including A, D, G and V) and anti-Kla amino acids (including C, L, Q, S and W). We also listed typical lactylation motifs, such as xxxxxxxxxx_K_xVxxxxxKxx. Since enzyme recognition of substrates depends to a large extent on adjacent amino acid sequences, we can deduce more “writers” and “erasers” beyond KATs, HDACs or SIRTs based on typical motifs.

Numerous histone lactylation sites were identified via this omics. Among the different histone types, the most significantly modified sites were H2AK95, H2BK46, H3K56, and H4K79. Specific target genes regulated by these sites can be identified through methods such as ChIP-seq and CUT-tag, and relevant genes related to repair healing can be screened.

### 3.3 Lactylation omics reveals metabolic relating therapy targets

Functional pathway enrichment revealed differentially modified proteins that covers a wide range of processes, such as glycolysis, TCA cycle, RNA processing and DNA processing. Combining 5d and 5w group, we found consistently up-regulated pathways such as RNA and DNA processes, as well as consistently down-regulated pathways such as glycolysis, lipid and nucleotide metabolism. These pathways directly interface with the metabolic demands of rotator cuff repair, where energy allocation, gene expression, and nutrient transport should be tightly coordinated., aligning with epigenetic regulation and metabolic reprogramming function of lactylation.

In “RNA processing” of 5d group, significant increased lactylation level of RNA helicase is notable. RNA helicases are ATP-dependent enzymes that govern critical RNA metabolic steps: translation initiation, ribosome biogenesis, precursor mRNA splicing, and mRNA decay (49, 50). During repair, lactylation of RNA helicase might modulates its activity to enhance helicase activity so as to stabilize mRNAs encoding or resolve secondary structures to facilitate translation. This positions RNA helicase as a metabolic sensor whose lactylation status may adapt RNA metabolism to the energy-limited, pro-inflammatory environment of early repair.

In “transport” of 5d group, significant decreases of lactylation in transferrin and hemoglobin were detected. Tendons are inherently ischemic and hypoxic (51), creating a challenge for repair due to lacking of nutrients including iron and oxygen. Transferrin plays a role in promoting cartilage growth (52) and craniofacial morphogenesis (53, 54), while hemoglobin is crucial for adaptation to hypoxia (55). We propose that decreased lactylation enhances their transport efficiency or capacity, which ensures adequate nutrient supply during the initial inflammatory phase when metabolic demand peaks—a critical link between metabolic support and repair initiation.

The “cellular adhesion” of 5w group were significantly up-regulated, including integrins, fibronectin, cadherins and focal adhesion proteins. The 5th week marks the proliferation and remodeling phase of the tendon healing process, where tenocytes and fibroblasts require robust cell-extracellular matrix interactions to establish tissue architecture. Integrins can activate PI3K/Akt signaling to drive cytoskeletal rearrangement and cell migration (53). Fibronectin acts as a provisional scaffold, templating ordered collagen fibrillogenesis and crosslinking monomers into mature fibers (56). Therefore, lactylation of these connection proteins may aid in remodelingfibers and enhancing cell-adhesive properties after rotator cuff tear, thus facilitating healing process.

### 3.4 Issues to be addressed

The samples analyzed in this lactylation modification omics were from the entire rotator cuff tissue. Therefore, the results cannot distinguish between intracellular and extracellular components, nor can they specifically focus on a particular cell type. Tendon tissue repair relies on the proliferation and secretion of extracellular matrix by myofibroblasts (namely tenocytes), clearance of necrotic tissue and release of cytokines by macrophages, as well as assistance from plasma cells, red blood cells, and others. Therefore, omics methods require further subdivision of each individual cell type, because analyzing only whole tissue is far from sufficient.

Both the “glycolysis” and “mitochondrial processes” categories showed significant lactylation changes in both 5d and 5w groups. Energy metabolism is crucial for cells, but the functional implications of lactylation-mediated metabolic reprogramming in tendon tissue are not yet clear. To repair torn rotator cuffs, cells require more carbon sources, so energy metabolism may shift from OXPHOS to glycolysis to provide abundant three-carbon compounds. However, the repair process inevitably requires higher levels of OXPHOS to provide more ATP. This situation creates a contradiction.

## 4 Experimental methods

### 4.1 Clinical samples

Human tendon samples were obtained from surgical patients in the Department of Sports Medicine, Peking University Third Hospital.

This project was approved by the Ethics Committee of Peking University Third Hospital, NO. S2024907.

### 4.2 Rabbit rotator cuff tear model

Incising the skin and subfascial layers along the lower edge of the acromion 1 cm and 0.5 cm posterior to the greater tuberosity. The deltoid muscle was dissected along the deltoid space, exposing the insertion point of the supraspinatus tendon, which was then cut with a blade along the bone surface with a width of approximately 5 mm to simulate rotator cuff injury. Finally, repair and fixation were performed using transosseous suturing.

This project was approved by the Ethics Committee of Peking University Third Hospital, NO. S20250417.

### 4.3 Lactate content measurement

According to the manufacturer’s instructions (Lactate kit, Solarbio, BC2230), we homogenized samples for 5 min in grinder, and then measured lactate content by standard colorimetric methods.

### 4.4 Western blot

Proteins from tendon samples were lysed and collected using RIPA lysis buffer containing proteinase inhibitors and phosphatase inhibitors. The protein concentration was determined by the BCA method and adjusted to the same final concentration. After boiling with loading buffer for denaturation, equal amounts of protein were loaded onto SDS–PAGE gels and electrophoresed at 140 mV for 1 h. The proteins were then transferred to PVDF membranes. The membranes were blocked with BSA at room temperature for 2 h, washed 3 times with TBST, and then incubated with primary antibodies overnight at 4 °C. After washing 3 times with TBST, the membranes were incubated with secondary antibodies at room temperature for 1 h, washed again 3 times with TBST, and finally detected using enhanced chemiluminescence (ECL).

### 4.5 Immunofluorescence

Tendon samples were fixed in 4% paraformaldehyde, dehydrated, embedded, and sectioned. Antigen retrieval was performed with pepsin for 1 h, followed by washing with PBST containing Tween-20 for 15 min and blocking with goat serum for 1 h. Primary antibodies were diluted at a ratio of 1:200 and incubated overnight at 4 °C. After washing 3 times with PBST, the fluorescent secondary antibodies were diluted at a ratio of 1:200 and incubated at room temperature in the dark for 1 h. After washing 6 times with PBST, DAPI staining was performed at room temperature for 5 min. After washing 3 times with PBST, the excess liquid was removed, and the coverslips were mounted. Finally, images were captured using a confocal microscope.

### 4.6 Lactylation omics

Collecting rotator cuff samples of NM, 5d and 5w groups (*n* = 3) for lactylation omics detection.

#### 4.6.1 Tissue processing before sample loading

Protein extraction started by taking samples from −80 °C, transferring an appropriate amount to a pre - cooled liquid nitrogen mortar and grinding with liquid nitrogen into powder. The samples were then mixed with 4 - volume lysis buffer and sonicated, followed by centrifugation at 4 °C, 12000 g for 10 min to collect the supernatant for protein concentration measurement using the BCA kit. Equal - amount protein samples were enzymatically digested. After volume adjustment with lysis buffer, pre - cooled acetone was added for precipitation at −20 °C for 2 h, then centrifuged at 4500 g for 5 min. The precipitate was washed with acetone, dried, and resuspended in 200 mM TEAB by sonication, followed by overnight incubation with trypsin at a 1:50 ratio (m/m). The sample was reduced with 5 mM DTT at 37 °C for 60 min and alkylated with 11 mM IAM in the dark at room temperature for 45 min. Another trypsin digestion was done at a 1:100 ratio (m/m) at 37 °C for 4 h. The hydrolyzed peptides were acidified to pH 2–3 with 10% TFA, centrifuged at 12000 g for 10 min at room temperature, and the supernatant was transferred. After activating the SPE column with methanol and balancing with 0.1% TFA, the acidified peptide solution was loaded, desalted with 0.1% TFA, and eluted with 80% ACN. Finally, 2uL of the peptide sample was taken for concentration measurement using a Nanophotometer NP80, with the UV protein measurement mode, adjusting OD to 1, blank - calibrating with a solvent sample, and then measuring the sample.

#### 4.6.2 LC–MS/MS analysis

The tryptic peptides were dissolved in solvent A, directly loaded onto a home-made reversed-phase analytical column (25-cm length, 100 μm i.d.). The mobile phase consisted of solvent A (0.1% formic acid, 2% acetonitrile/in water) and solvent B (0.1% formic acid in acetonitrile). Peptides were separated with following gradient: 0–42 min, 9-25%B; 42–52 min, 25-35%B; 52–56 min, 35-90%B; 56–60 min, 90%B, and all at a constant flow rate of 450 nL/min on a Easy-nLC1000. The peptides were subjected to capillary source followed by the timsTOF Pro mass spectrometry. The electrospray voltage applied was 1.65 kV. Precursors and fragments were analyzed at the TOF detector, with a MS/MS scan range from 100–1700. The timsTOF Pro was operated in parallel accumulation serial fragmentation (PASEF) mode. Precursors with charge states 0–5 were selected for fragmentation, and 10PASEF-MS/MS scans were acquired per cycle. The dynamic exclusion was set to 24 s.

#### 4.6.3 Pan antibody-based PTM enrichment

To enrich modified peptides, tryptic peptides dissolved in NETN buffer (100 mM NaCl, 1 mM EDTA, 50 mM Tris–HCl, 0.5% NP-40, pH 8.0) were incubated with pre-washed antibody beads (Lot number PTM1404, PTM Bio) at 4 °C overnight with gentle shaking. Then the beads were washed for four times with NETN buffer and twice with H2O. The bound peptides were eluted from the beads with 0.1% trifluoroacetic acid. Finally, the eluted fractions were combined and vacuum-dried. For LC–MS/MS analysis, the resulting peptides were desalted with C18 ZipTips (Millipore) according to the manufacturer’s instructions.

#### 4.6.4 Database retrieval

The resulting MS/MS data were processed using the MaxQuant search engine (v.1.6.15.0). Tandem mass spectra were searched against the rabbit SwissProt database (20,422 entries) concatenated with the reverse decoy database. Trypsin/P was specified as a cleavage enzyme allowing up to 2 missing cleavages. The mass tolerance for precursor ions was set as 20 ppm in the first search and 5 ppm in the main search, and the mass tolerance for fragment ions was set as 0.02 Da. Carbamidomethyl on Cys was specified as a fixed modification, and acetylation on the protein N-terminus and oxidation on Met were specified as variable modifications. The FDR was adjusted to < 1%.

#### 4.6.5 Subcellular localization

The proteins in the cells of eukaryotic tissues are located on various elements in the cell based on the difference of the membrane structure that they bind to. The main subcellular locations of eukaryotic cells include: extracellular, cytoplasm, nucleus, mitochondrion, Golgi apparatus, endoplasmic reticulum, peroxisome, vacuole, cytoskeleton, nucleoplasm, nuclear matrix and ribosome. Thus, we annotated the subcellular structure of the protein using WoLF PSORT software.

#### 4.6.6 GO enrichment

GO annotations of proteins are divided into three broad categories: Biological Process, Cellular Component, and Molecular Function. Fisher’s exact test was used to analyze the significance of GO enrichment of differential proteins (using the identified protein as the background), and a *p* value <0.05 was considered to indicate statistical significance.

#### 4.6.7 KEGG enrichment

The Kyoto Encyclopedia of Genes and Genomes (KEGG) database was used for KEGG pathway enrichment analysis. Fisher’s exact test was used to analyze the significance of KEGG pathway enrichment of differential proteins (using the identified protein as the background), and a *p* value <0.05 was considered to indicate statistical significance.

### 4.7 Quantification and statistical analysis

All data are expressed as mean ± standard error of the mean (SEM) unless otherwise described. Statistical analyses were performed using GraphPad Prism (version 9.0) and the significance of differences was assessed by two tail unpaired Student’s t test or one-way or two-way analysis of variance (ANOVA). Significance was set as *p* < 0.05 and expressed as *p < 0.05, **p < 0.01, and ***p < 0.001.

## Data Availability

The data presented in the study are deposited in the ProteomeXchange repository, accession number PXD062718.

## Appendix A: Reagents

**Table:**

Name	Company	Cat no.
Triton X-100	Sangon Biotech	A110694-0100
Protease Inhibitor Cocktail III	Merck Millipore	539134-10ML
TSA (trichostatin A)	MedChemExpress	HY-15144
NAM (nicotinamide)	Sigma-Aldrich	N0636-500g
BCA kit	Beyotime Biotechnology	P0011
Triethylammonium bicarbonate buffer (TEAB)	Sigma-Aldrich	T7408-500mL
Trypsin	Promega	V5117
Dithiothreitol (DTT)	Sigma-Aldrich	D9163-25G
iodoacetamide (IAM)	Sigma-Aldrich	V900335-5G
Acetonitrile	ThermoFisher Scientific	204433
Trifluoroacetic acid (TFA)	Sigma-Aldrich	302031-1L
Methanol	ThermoFisher Scientific	A452-4
SPE strata-X100mg/3 mL	phenomenex	8B-S100-EBJ
4% Polyformaldehyde	Biosharp	BL539A
Rabbit polyclonal anti-pan Kla	PTM BIO	PTM-1401
Mouse monoclonal anti-alpha tubulin	PTM BIO	PTM-5001
Donkey Anti-Rabbit	Abcam	ab205722
Donkey Anti-Rabbit	Abcam	ab150073
Goat Serum	BOSTER	AR0009
Lactate kit	Solarbio	BC2230

## Glossary

ACSS2: Acetyl-CoA Synthetase 2
AARS: Alanyl-tRNA Synthetase
Arg1: Arginase 1
cGAS: Cyclic GMP-AMP Synthase
ChIP-seq: Chromatin Immunoprecipitation sequencing
CUT-Tag: Cleavage under targets and tagmentation
DCA: Sodium dichloroacetate
GO: Gene ontology
GTPSCS: GTP-dependent succinyl-CoA synthetase
HDAC: Histone deacetylase
IF: Immunofluorescence
KAT: Lysine acetyltransferase
KEGG: Kyoto Encyclopedia of Genes and Genomes
Kla: Lysine lactylation
LDH: Lactate dehydrogenase
LGSH: Lactoylglutathione
LPS: Lipopolysaccharide
MCT: Monocarboxylate transporter
MTS: Modification tendency suppression
MTE: Modification tendency enhancement
OXPHOS: Oxidative phosphorylation
PTM: Post-translational modification
PKM: Pyruvate kinase
sMPN: Significant modification propensity neglect
sMPP: Significant modification propensity preference
SIRT: Sirtuin
VEGF: Vascular endothelial growth factor
