# Supplementary material for: Lactylation omics of rabbit rotator cuff tear reveals differentially modified proteins and metabolic relating therapy targets
Source: Front Med (Lausanne). 2026 Mar 17;13:1797466. doi: 10.3389/fmed.2026.1797466 (PMC13037487; doi:10.3389/fmed.2026.1797466)
Supplement: Supplementary file 1 [file Table_1.docx]

**Table S1: Differential protein function enrichment: 5d vs NM**

|  | up-regulated | | down-regulated | | |
| --- | --- | --- | --- | --- | --- |
|  | classifications | proteins | classifications | | proteins |
| GO | **RNA**  **processing** | coronin  stathmin  nucleolin  nucleophosmin  RNA helicase  interleukin enhancer binding factor 3  BCL2 associated transcription factor 1  heterogeneous nuclear ribonucleoprotein  eukaryotic translation initiation factor 4  Ppoline rich mitotic checkpoint control factor  activated RNA polymerase II transcriptional coactivator p15 | **ATP metabolism**  **＆**  **mitochondrial**  **processing** | ATP synthase  aconitate hydratase  enoyl-CoA hydratase  ADP/ATP translocase  NADH dehydrogenase  AMP phosphotransferase  enoyl-CoA delta isomerase 1  calcium-transporting ATPase  phosphopyruvate hydratase  cytochrome c oxidase subunit 4  acetyl-CoA acetyltransferase 1  phosphoenolpyruvate carboxykinase  acyl-CoA synthetase family member 2 | |
|  | **DNA**  **processing** | CSRP1  chromobox 5  yhymosin beta  protein S100-A  histone deacetylase 1  alpha-2-macroglobulin  nuclear receptor corepressor 1  chromo domain-containing protein  KAT8 regulatory NSL complex subunit  chromosome 17 open reading frame 49  metastasis associated 1 family member 2  apoptotic chromatin condensation inducer 1  thyroid hormone receptor associated protein 3 | **myosin** | actin  troponin  vimentin  myomesin  nebulette  myosin binding protein C | |
|  |  |  | **pyruvate**  **metabolism** | pyruvate kinase  lactate dehydrogenase  pyruvate dehydrogenase complex | |
|  |  |  | **transporting** | transferrin  annexin  reticulon  hemoglobin  actinin alpha  apolipoprotein A  anion exchange protein  calcium-transporting ATPase  aspartate aminotransferase  fatty acid-binding protein  60 kDa heat shock protein  high mobility group protein B1 | |
| KEGG | **spliceosome** | RNA helicase  heterogeneous nuclear ribonucleoprotein K  apoptotic chromatin condensation inducer 1 | **glycolysis / gluconeogenesis** | beta-enolase  pyruvate kinase  phosphoglycerate mutase  6-Phosphofructokinase  7-Phosphopyruvate hydratase  phosphoglycerate kinase  fructose-bisphosphate aldolase  triosephosphate isomerase  glucose-6-phosphate isomerase  phosphoenolpyruvate carboxykinase  glyceraldehyde-3-phosphate dehydrogenase | |
|  | **other** | transferrin  apolipoprotein A  histone deacetylase 1  nuclear receptor corepressor 1 | **TCA cycle** | isocitrate dehydrogenase  malate dehydrogenase  citrate synthase | |
|  |  |  | **OXPHOS** | ATP synthase  NADH dehydrogenase  cytochrome c oxidase subunit 4 | |
|  |  |  | **lipid metabolism** | apolipoprotein A  aspartate aminotransferase  enoyl-CoA hydratase  acetyl-CoA acetyltransferase  hydroxyacyl-CoA dehydrogenase | |
|  |  |  | **pyruvate metabolism** | lactate dehydrogenase  pyruvate dehydrogenase complex  acetyl-CoA acetyltransferase | |
|  |  |  | **PI3K-Akt**  **signaling pathway** | collagen alpha-1(I) chain  collagen alpha-3(VI) chain  immunoglobulin heavy constant mu  tyrosine 3-monooxygenase/tryptophan 5-monooxygenase activation protein | |
