# Supplementary material for: Lactylation omics of rabbit rotator cuff tear reveals differentially modified proteins and metabolic relating therapy targets
Source: Front Med (Lausanne). 2026 Mar 17;13:1797466. doi: 10.3389/fmed.2026.1797466 (PMC13037487; doi:10.3389/fmed.2026.1797466)
Supplement: Supplementary file 2 [file Table_2.docx]

**Table S2: Differential protein function enrichment: 5w vs NM**

|  | up-regulated | | down-regulated | |
| --- | --- | --- | --- | --- |
|  | classifications | proteins | classifications | proteins |
| **GO** | **cotranslational protein targeting to membrane** | ribosomal protein L24  40S ribosomal protein S12  60S ribosomal protein L29  signal recognition particle subunit SRP68 | **nucleotide metabolism** | alpha-synuclein  adenosine kinase  AMP phosphotransferase  methyl-CpG-binding protein 2  adenylate kinase isoenzyme 1 |
|  | **protein localization to mitochondrion** | calmodulin  60 kDa heat shock protein  ubiquinol-cytochrome c reductase core protein 2 | **regulation of actin filament length** | destrin  cortactin  actin-α |
|  | **protein localization to nucleus** | lamin  cofilin  calreticulin  pre-mRNA-splicing factor RBM22 | **other** | moesin  hemoglobin  glutaredoxin  ATP synthase  aminopeptidase  glycogen phosphorylase  heterogeneous nuclear ribonucleoprotein |
|  | **cell adhesion** | integrin  cadherin  vinculin  cortactin  fibronectin |  |  |
|  | **wound healing** | S-100 A9  vimentin  hemoglobin  filamin  myoferlin  kininogen  annexin  vitronectin  plasminogen  fibrinogen  tropomyosin  antithrombin  beta-2-glycoprotein  alpha-2-macroglobulin  complement C3 alpha chain  macrophage metalloelastase  cellular communication network factor 1 |  |  |
| **KEGG** | **ribosome** | ribonucloprotein  40S ribosomal protein  60S ribosomal protein  NOP58 ribonucleoprotein  GTP-binding nuclear protein Ran  dyskerin pseudouridine synthase 1 | **glycolysis /**  **gluconeogenesis** | beta-enolase  phosphoglycerate mutass  phosphoglycerate kinase  aldehyde dehydrogenase  6-phosphofructokinase  fructose-bisphosphate aldolase  pyruvate dehydrogenase complex  phosphoenolpyruvate carboxykinase |
|  | **spliceosome** | RNA helicase  ransformer 2 beta homolog  Pre-mRNA-splicing factor RBM22  Apoptotic chromatin condensation inducer 1  Heterogeneous nuclear ribonucleoprotein K  Heat shock protein family A (Hsp70) member 8 | **TCA cycle** | citrate synthase  aconitate hydratas  fumarate hydratase  succinate-CoA ligase  malate dehydrogenase  succinate dehydrogenase  isocitrate dehydrogenase |
|  | **protein precessing** | endoplasmin  Calreticulin  NSFL1 cofactor p4  Hypoxia up-regulated 1  Protein disulfide-isomerase  Ribosome binding protein 1  Heat shock protein HSP 90-alpha  B-cell receptor-associated protein  Cytoskeleton associated protein 4 | **amino acid**  **metabolism** | sulfurtransferase  aspartate aminotransferase  hydroxyacyl-CoA dehydrogenase  methylcrotonoyl-CoA carboxylase |
|  |  |  | **lipid metabolism** | apolipoprotein A  hydroxysteroid 17-beta dehydrogenase |
|  |  |  | **HIF-1 signaling pathway** | beta-enolase  6-phosphofructokinase  phosphoglycerate kinase  fructose-bisphosphate aldolase A、B、C |
|  |  |  | **other** | carbonic anhydrase 2  anion exchange protein  glycogen phosphorylase  calcium-transporting ATPase  glutathione S-transferase kappa 1 |
